# Correlation of prefrontal cortical activation with changing vehicle speeds in actual driving: a vector-based functional near-infrared spectroscopy study

**DOI:** 10.3389/fnhum.2013.00895

**Published:** 2013-12-25

**Authors:** Kayoko Yoshino, Noriyuki Oka, Kouji Yamamoto, Hideki Takahashi, Toshinori Kato

**Affiliations:** ^1^Department of Brain Environmental Research, KatoBrain Co., Ltd.Tokyo, Japan; ^2^Department of Environment/Engineering, Tokyo Branch, Central Nippon Expressway Co., Ltd.Tokyo, Japan; ^3^Department of Environment/Engineering, Central Nippon Expressway Co., Ltd.Nagoya, Japan

**Keywords:** actual driving, supplementary eye field, outdoor brain activation, acceleration, deceleration, interregional correlation, phase angle, vehicle acceleration

## Abstract

Traffic accidents occur more frequently during deceleration than during acceleration. However, little is known about the relationship between brain activation and vehicle acceleration because it has been difficult to measure the brain activation of drivers while they drive. In this study, we measured brain activation during actual driving using vector-based functional near-infrared spectroscopy. Subjects decelerated from 100 to 50 km/h (speed reduction task) and accelerated from 50 to 100 km/h (speed increase task) while driving on an expressway, in the daytime and at night. We examined correlations between average vehicle acceleration in each task and five hemodynamic indices: changes in oxygenated hemoglobin (ΔoxyHb), deoxygenated hemoglobin (ΔdeoxyHb), cerebral blood volume (ΔCBV), and cerebral oxygen exchange (ΔCOE); and the phase angle *k* (degrees) derived from the other hemoglobin (Hb) indices. ΔoxyHb and ΔCBV reflect changes in cerebral blood flow, whereas ΔdeoxyHb, ΔCOE, and *k* are related to variations in cerebral oxygen metabolism. Most of the resulting correlations with specific brain sites, for all the indices, appeared during deceleration rather than during acceleration. Faster deceleration resulted in greater increases in ΔdeoxyHb, ΔCOE, and *k* in the prefrontal cortex (*r* < −0.5, *p* < 0.01), in particular, in the frontal eye field, and at night, it also resulted in greater decreases in ΔoxyHb and ΔCBV in the prefrontal cortex and in the parietal lobe (*r* > 0.4, *p* < 0.01), suggesting oxygen metabolism associated with transient ischemic changes. Our results suggest that vehicle deceleration requires more brain activation, focused in the prefrontal cortex, than does acceleration. From the standpoint of the indices used, we found that simultaneous analysis of multiple hemodynamic indices was able to detect not only the blood flow components of hemodynamic responses, but also more localized frontal lobe activation involving oxygen metabolism.

## Introduction

In recent years, neuroscience research related to vehicle driving has become popular. Brain function during driving encompasses responses to external stimuli such as visual stimulation and vehicle acceleration, in addition to internal processing involved in functions such as motor control and decision making. Functional neuroimaging during driving examines multiple intricate factors such as perception, cognition, thinking, and motor functions. It is important for road safety measures in the Intelligent Transport Systems (ITS) field to separate these factors and identify the regions of interest in the cerebral cortex for each of them.

Kato et al. (2013) and Yoshino et al. (2013) demonstrated that areas of the brain that are activated during acceleration were significantly different from those activated during deceleration in actual road experiments. That study suggested that brain activation during deceleration is higher than activation during acceleration; and this led us to investigate in more detail the relationship between brain activity and vehicle acceleration. The most common cause of traffic accidents between vehicles in Japan is the rear-end collision (approximately 38.5%) (Institute for Traffic Accident Research and Data Analysis, 2011). Rear-end collisions are caused by deceleration that occurs too late. It is possible that this is related to differences in brain function between acceleration and deceleration. However, no studies have yet examined brain responses during vehicle acceleration and deceleration in actual road driving.

Since there has been very little investigation of brain activity during actual road driving, the kind of responses that can be expected is still unclear. We thus decided to use fNIRS vector-based analysis, which is capable of explaining all the possible variations in the ratios of concentration changes in oxyHb and deoxyHb (Kato, 2006, 2007). This is a method that displays compositely the hemodynamic response dues to changes in both oxyHb and deoxyHb on the same vector coordinate plane. Oxygenation and blood volume changes reflecting neural activity cannot be properly evaluated using the conventional analysis of either oxyHb or deoxyHb alone. In vector-based analysis, a cerebral blood volume (CBV) axis and a cerebral oxygen exchange (COE) axis are generated from an oxyHb and deoxyHb orthogonal coordinate plane, and the phase of vectors on this vector plane are evaluated (Yoshino and Kato, 2012; Sano et al., 2013), increasing the possible indices of brain activity. We thought it would be possible to use this method to evaluate brain activity during actual expressway driving from various perspectives.

We therefore investigated the relationship between brain regions and hemodynamic indices that increased or decreased in relation to calculated vehicle acceleration (m/s^2^) during tasks, using vector-based functional near-infrared spectroscopy (fNIRS) in a vehicle in an actual road experiment. This study aimed to extract brain responses and indices that are related to vehicle acceleration and deceleration by recording changes in cortical hemodynamic responses during driving by normal adults both during daytime and at night.

## Methods

### Subjects

Right-handed twelve healthy adults participated in this study (eight males and four females; average age, 33.3 ± 4.5 years). The subjects had operational experience on expressways and ordinary roads on a daily basis. The subjects had no history of mental or central nervous illnesses, and they took no medications on the day of the experiment. The subjects were comfortable in the experimental situation because they had previously participated in other actual expressway driving research (Yoshino et al., 2013) while wearing the fNIRS system probes. Written consent was obtained from the participants before enrollment in the study, and the protocol was approved in advance by the ethics committee at KatoBrain Co., Ltd. The subjects' average length of driving history was 11.8 ± 5.8 years. Their frequency of driving was 6.1 ± 1.6 times / week, and their frequency of expressway driving was 4.5 ± 6.5 times / month. Only two subjects had experienced an accident (neither accident involved another vehicle or any personal injuries), and the average number of accidents was 0.2 ± 0.4. The average number of traffic violations was 1.3 ± 1.3 times, mostly for speeding. Since recruitment of the subjects was based on the conditions of age, right-handedness, and frequency of driving on a daily basis, the subjects' genders, their driving histories, and their histories of violations and accidents were completely random.

### Experimental field and test vehicle

The experiment was performed in the Okitsu district, Shizuoka Prefecture, Japan, on a section of the Shin Tomei Expressway immediately before it entered service (Yamamoto et al., 2012; Kato et al., 2013). Installation of signage, lighting and so on had already been completed, and so there were no problems with the safety of vehicle travel. For further safety, no vehicles were present other than the test vehicle on the experimental course. Guard personnel were located at each point on the experimental course, and they could immediately contact the test vehicle with a transceiver in any unexpected contingencies.

The experimental course was 2875 m long. It was almost straight, but included a gentle left and right curve (*R* = 5000, both). The slope of the course was almost flat, but there were uphill and downhill gradients of 2.0%. The test vehicle traveled in the left lane (3.75 m in width) in accordance with Japanese traffic regulations for lane use in two lanes. There was no artificial lighting provided on the experimental course, either in the daytime or at night.

An ordinary van, *Hiace*, which is made by the Toyota Motor Corporation (Japan) and is super-long with a high-roof specification, was used in this experiment. The vehicle was a two-wheel-drive, gasoline-powered vehicle with a four-speed automatic transmission. A global positioning system receiver and a vehicle speed pulse counter were attached to the test vehicle, to record information on vehicle position, speed, and acceleration. Power was supplied to the fNIRS equipment by connecting a DC/AC inverter to the battery of the vehicle.

### Experimental procedure

**Figure 1:**
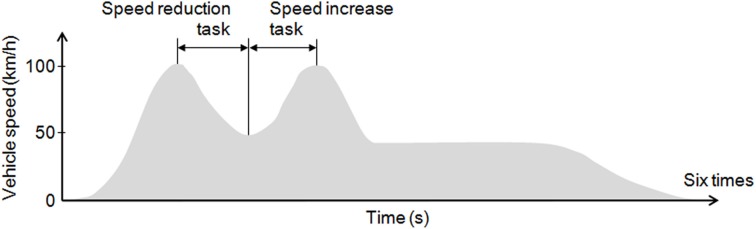
**Experimental tasks**. Each subject performed 6 daytime trials and 6 nighttime trials on the experimental course. The tasks included a speed reduction task of deceleration from 100 to 50 km/h and a speed increase task of acceleration from 50 to 100 km/h.

The tasks included a speed reduction task of deceleration from 100 to 50 km/h and a speed increase task of acceleration from 50 to 100 km/h. The speed increase task was performed immediately after the speed reduction task (Figure 1). One trial consisted of these two driving tasks, and six trials each were performed in the daytime and at night. Start and stop positions were provided on the course, and the subjects performed the two tasks at their own pace, with no cues on the course. After receiving their instructions and before putting on the fNIRS probes, the subjects performed 1–3 practice drives for the daytime and nighttime trials. The day and night experiments were performed on different days and the order of the experiments (day or night) was randomized between the subjects. The average duration of the speed increase task (acceleration) was 16.8 ± 3.3 s, and the average duration of the speed reduction task (deceleration) was 21.1 ± 8.0 s. The average ratio of acceleration time to deceleration time for all the subjects was 0.90 ± 0.30.

### fNIRS measurements and registration

A multichannel fNIRS system (FOIRE-3000, Shimadzu Corporation, Japan) was mounted in the vehicle and used to measure hemodynamic responses. The equipment irradiated three wavelengths of NIR light (780, 805, and 830 nm) to the cerebral cortex, and monitored changes in the hemoglobin (Hb) concentrations. Sampling intervals for measuring changes in Hb concentration were set to 70 ms.

The fNIRS device was tightly secured to the vehicle using two bars installed behind the driver's seat and a hook on the floor of the vehicle. The probe line was also attached to the bars behind the driver's seat. Probes were attached to the subject's head in a way that allowed for moderate changes in driving posture. To prevent noise due to sunlight, the front and rear of the device, and the subject's head were covered with black cloth after the probes were attached.

**Figure 2:**
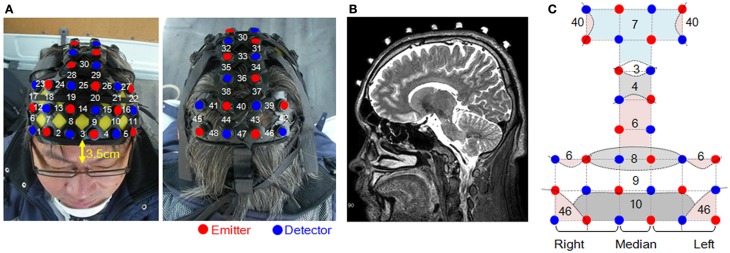
**Measurement sites and the locations of the probes. (A)** Head probe attachments and channel numbers. **(B)** Positioning of the probes was confirmed using MRI. **(C)** Dashed curves represent the schematic boundaries of the Brodmann areas that were confirmed by MRI. Straight lines connecting the emitters and detectors indicate channel positions.

Measurement areas were located on both sides of the prefrontal cortex, and on the motor cortex and the parietal cortex; the occipital lobe was excluded for the safety of the subjects (Figure 2A). Forty-eight channels were set up using 16 irradiation and 16 detection probes. The distance between irradiation and detection probes was 3 cm.

Confirmation of the position of each measurement point was done by magnetic resonance imaging (MRI), using a 3-Tesla 3D-T2-weighted MRI system (Philips Co., Achieva 3.0 Quasar Dual 3.0T-MRI), in which the subject probe attachments were fitted with registration markers. The sampling conditions used the spin-echo method with an echo time of 247 ms, a repetition time of 2700 ms, image size of 250 × 250 pixels, a slice thickness of 1.0 mm in the sagittal direction, and an interslice gap of 0 mm. As Figure 2B shows, the positions of the probes were confirmed for all of the measured areas based on the locations of the registration markers. Figure 2C shows the Brodmann areas (BA) having the highest correspondence with each channel, from the MRIs of all the subjects.

### Analysis

#### The vector approach

In the blood vessels, changes in oxygenation and blood volume occur in response to neural activity, and changes in the concentrations of both oxyHb and deoxyHb are involved in this response. To detect changes in both oxygenation and blood volume, the following vector analysis method was used.

**Figure 3:**
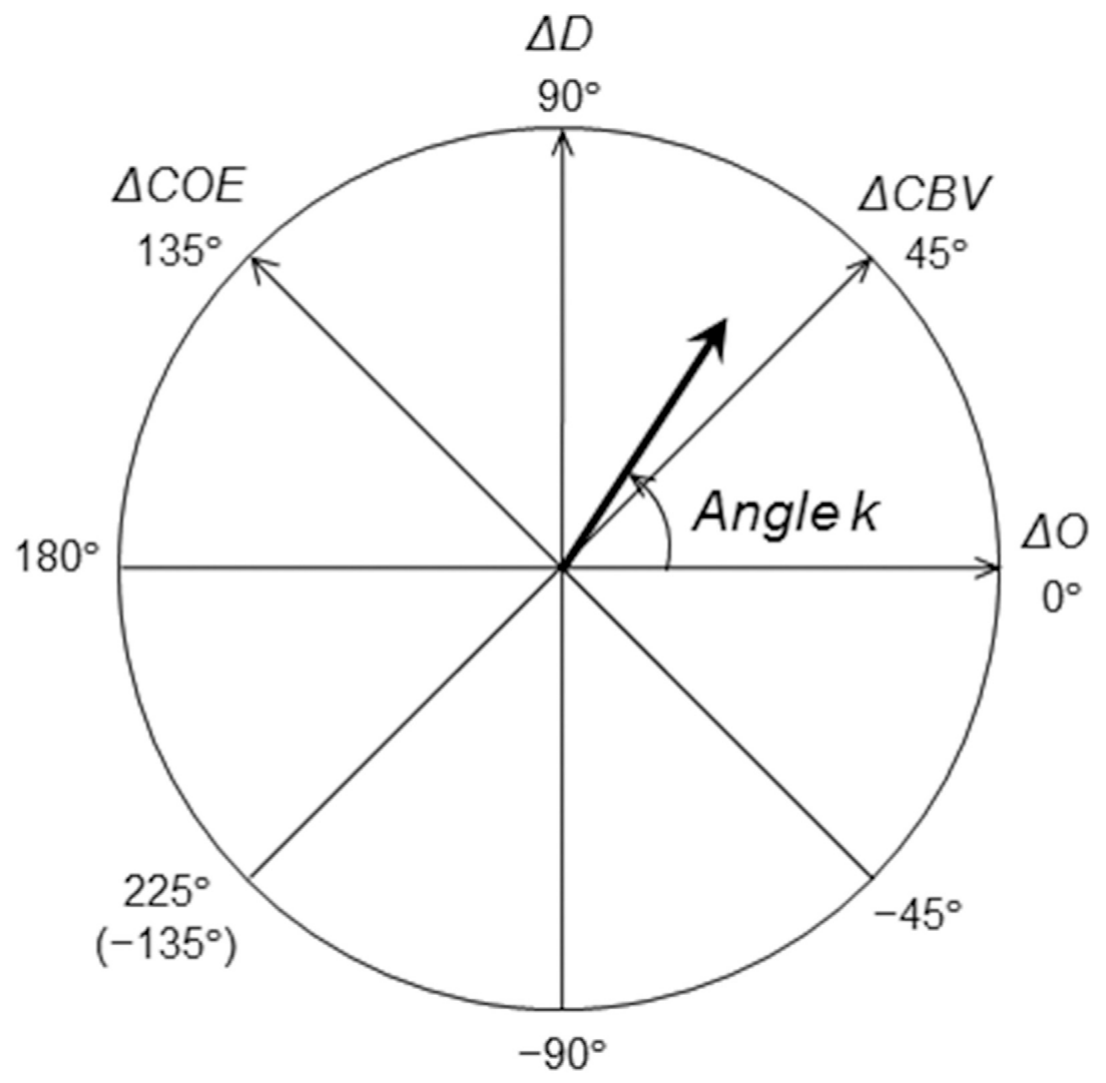
**Definition of the vector coordinates**. Polar coordinate plane for the analysis of cerebral oxygenation and blood volume. The relationship between cerebral oxygen exchange (ΔCOE) and cerebral blood volume (ΔCBV) can be detected by vector trajectories.

OxyHb and deoxyHb have different chemical properties (paramagnetic or diamagnetic) that are due to differences in the bonding of oxygen molecules (Pauling and Coryell, 1936). Taking this into consideration, an orthogonal vector coordinate plane is set up, defined by oxyHb (Δ*O*) and deoxyHb (Δ*D*) axes (Kato, 2006, 2007). As Figure 3 shows, rotating this Δ*O*/Δ*D* vector plane 45 degrees counterclockwise results in an orthogonal vector coordinate plane comprising a (Δ*O* + Δ*D*) axis and a (Δ*D* − Δ*O*) axis. A vector (Δ*O* + Δ*D*) can be defined as a cerebral blood volume (ΔCBV) vector, and a vector (Δ*D* − Δ*O*) can be defined as a cerebral oxygen exchange vector (ΔCOE). A positive value for ΔCBV indicates locally increasing ΔCBV and a negative value for ΔCBV indicates decreasing ΔCBV. A positive value of ΔCOE indicates hypoxic change from ΔCOE = 0, and a negative value of ΔCOE indicates hyperoxic change. The relationship among the four axes of Δ*O*, Δ*D*, ΔCBV, and ΔCOE is described by the following square matrix:

(1) $$\left(\begin{array}{l} \Delta O+\Delta D\\ -\Delta O+\Delta D \end{array}\right)=\left(\begin{array}{ll} 1 & 1\\ -1 & 1 \end{array}\right)\left(\begin{array}{l} \Delta O\\ \Delta D \end{array}\right)=\left(\begin{array}{l} \Delta \text{CBV}\\ \Delta \text{COE} \end{array}\right)$$

(2) $$\left(\begin{array}{l} \Delta O\\ \Delta D \end{array}\right)=\frac{1}{2}\left(\begin{array}{ll} 1 & -1\\ 1 & 1 \end{array}\right)\left(\begin{array}{l} \Delta \text{CBV}\\ \Delta \text{COE} \end{array}\right)$$

These can be expanded to obtain ΔCBV and ΔCOE as follows.

(3) $$\Delta \text{CBV}=\frac{\left(\Delta D+\Delta O\right)}{\sqrt{2}}$$

(4) $$\Delta \text{COE}=\frac{\left(\Delta D-\Delta O\right)}{\sqrt{2}}$$

The phases (octants) on the vector plane described above provide a quantitatively defined representation of the degree of oxygen exchange; the phase of a vector is defined by the angle *k* (degrees), which is a ratio of ΔD to ΔO, and it reflects the strength of oxygen metabolism. *k* is the angle between a vector and the positive ΔO axis, and it is determined as follows:

(5) $$\begin{array}{l} k=Arc\tan\left(\frac{\Delta D}{\Delta O}\right)\\ \;=Arc\tan\left(\frac{\Delta \text{COE}}{\Delta \text{CBV}}\right)+\;45^{{}^{\circ}}\;(-135^{{}^{\circ}}\;≦\;k\;≦\;225^{{}^{\circ}}) \end{array}$$

*k* = 0° is on the positive Δ*O* axis, and coincides with the oxygen density of arterial blood. An increase in *k* is defined within the range of increase in Δ*D* or ΔCOE (0° ≦ *k* ≦ 235°), and a decrease in *k* is defined within the range of decrease in Δ*D* and ΔCOE (−135° ≦ *k* ≦ 0°). An increase or decrease in *k* thus indicates a change in oxygen demand.

#### Data processing and statistics

The Δ*D* and Δ*O* data were subjected to low-pass filtering at 0.1 Hz to remove any high frequency components. For each of these two indicators, the average changes per second among the tasks were determined for each channel. ΔCOE, ΔCBV, and *k* were calculated using these data. In this process, the actual changes for each task were calculated by setting the levels to zero at the beginning of each task.

Average vehicle acceleration (m/s^2^) during each task was determined by Equation (6). Positive values indicate average vehicle acceleration during speed increase tasks, and negative values indicate average vehicle acceleration during speed reduction tasks.

(6) $$\begin{array}{l} \text{Average vehicle acceleration}\\ \;=(\text{initial velocity }-\text{ final velocity})/\text{time} \end{array}$$

Analyses of correlations (Spearman's rank correlation coefficient) between average vehicle acceleration and each hemodynamic index were performed. Analyses of interregional correlations (Spearman's rank correlation coefficient) within each hemodynamic index were also performed. These tests were applied separately to each of the daytime and nighttime experiments. The data used for analysis was a total of 70 trials in the daytime and 70 trials in nighttime. Two trials each in the daytime and nighttime experiments were excluded because Hb monitoring was not successful. The significance level was set to 5%. Correlation coefficients that were higher than ±0.4 were evaluated.

## Results

### Average vehicle acceleration

Table 1 shows average vehicle acceleration during the two tasks. Negative values indicate deceleration. There were no significant differences between daytime and nighttime for either of the tasks.

**Table 1:** **Average vehicle acceleration**.

**Tasks**	**Vehicle acceleration (m/s^2^)**		***t***	***p***
	**Day time**	**Night time**		
Speed reduction task	−0.75 ± 0.27	−0.76 ± 0.30	0.182	0.856 (n.s.)
Speed increase task	0.86 ± 0.18	0.85 ± 0.16	0.291	0.772 (n.s.)

*n.s., not significant*.

### Correlations in the vehicle speed reduction task

**Figure 4:**
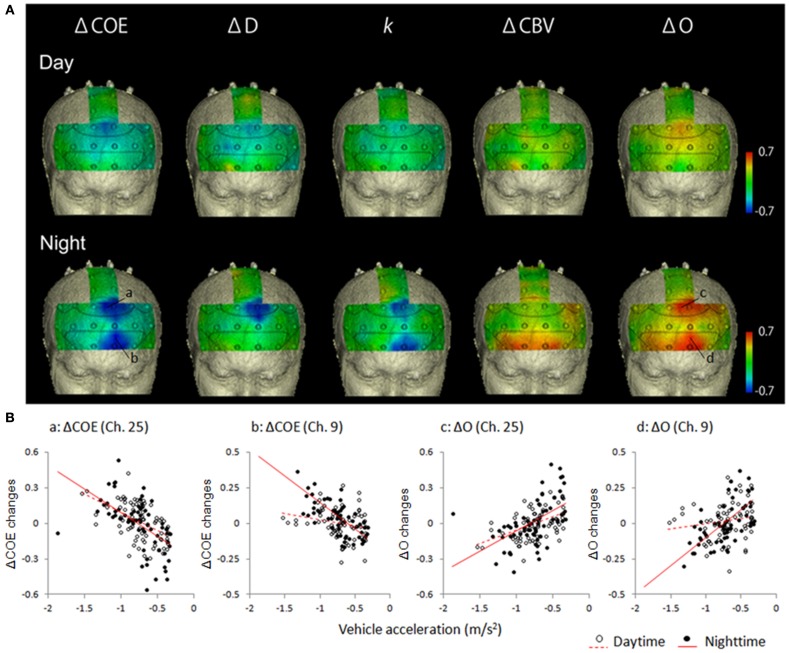
**Correlations in the speed reduction task between vehicle acceleration and each of the brain activation indices. (A)** Correlation maps showing correlations between vehicle acceleration and each of the hemodynamic indices in the speed reduction task. **(B)** Correlation diagrams from the medial BA8 (Ch. 25) and the left BA10 (Ch. 9) showed marked correlation with vehicle acceleration. Vehicle acceleration correlated negatively with ΔCOE and positively with Δ*O*. Correlation coefficients were higher at night than in the daytime.

Figure 4 shows mapping images of the correlations between average vehicle acceleration and each of the hemodynamic indices in the speed reduction task. The results divide into significant negative and positive correlations according to the indices used. Significant negative correlations were observed for ΔCOE, Δ*D*, and *k* (*p* < 0.01). Significant positive correlations were observed for ΔCBV and Δ*O* (*p* < 0.01).

#### Negative correlations in the vehicle speed reduction task

Table 2 shows correlations of −0.4 or lower in the speed reduction task. The greater the deceleration, the more ΔCOE, Δ*D*, and *k* increased. These correlations were observed only in the frontal lobe (BA10, BA9, BA8 and BA6), and none were observed in the parietal lobe.

**Table 2:** **Negative correlations in the speed reduction task between vehicle acceleration and the brain activation indices**.

		**Ch**.	**ΔCOE**		**Δ*D***		***k***	
			**Day**	**Night**	**Day**	**Night**	**Day**	**Night**
BA10	Med.	3		−0.604^*^				−0.597^*^
	Left	4		−0.663^*^				−0.614^*^
		9		−0.698^*^				−0.539^*^
BA9	Right	13			−0.493^*^			
	Left	20		−0.613^*^		−0.661^*^		−0.462^*^
BA8	Med.	25	−0.545^*^	−0.670^*^	−0.457^*^	−0.603^*^		−0.596^*^
	Left	26		−0.613^*^		−0.603^*^		−0.431^*^
BA6	Left	27	−0.413^*^		−0.424^*^			
		29	−0.408^*^	−0.622^*^		−0.543^*^		−0.540^*^

* *Correlations of r < −0.4 were all significant at p < 0.01*.

In the speed reduction task, high negative correlations were observed between ΔCOE and vehicle acceleration in the medial BA8 (daytime: *r* = −0.545, *p* < 0.001; nighttime: *r* = −0.670, *p* < 0.001). In the nighttime results, in addition to the negative correlations observed in the medial BA8, negative correlations increased in the peripheral regions of the left BA8 (Ch. 26), the left BA9 (Ch. 20), and the left BA6 (Ch. 29) (−0.622 < *r* < −0.613, *p* < 0.001). These correlations during the nighttime tasks were also observed with Δ*D* and *k* (Δ*D*: −0.661 < *r* < −0.543, *p* < 0.001; *k*: −0.596 < *r* < −0.431, *p* < 0.001). Negative correlations with vehicle acceleration were also observed with ΔCOE and *k* at night in the medial BA10 (Ch. 3) and in the left BA10 (Chs. 4 and 9) (ΔCOE: −0.698 < *r* < −0.604, *p* < 0.001; *k*: −0.614 < *r* < −0.539, *p* < 0.001).

#### Positive correlations in the speed reduction task

Table 3 shows correlations 0.4 or more in the speed reduction task. The greater the deceleration, the more ΔCBV and Δ*O* decreased. Activation occurred during the daytime tasks mainly on the periphery of the medial BA8 (*r* = 0.464, *p* < 0.001); and during the nighttime tasks, in the medial and the left BA8 and the area surrounding them (0.436 < *r* < 0.654, *p* < 0.001) and in both the right and left BA10 (0.486 < *r* < 0.674, *p* < 0.001). Positive correlations were also observed for ΔCBV in both the right and the left BA10 at night (0.422 < *r* < 0.601, *p* < 0.001).

**Table 3:** **Positive correlations in the speed reduction task between vehicle acceleration and each of the brain activation indices**.

		**Ch**.	**ΔCBV**		**Δ*O***	
			**Day**	**Night**	**Day**	**Night**
BA10	Right	2	0.434^*^	0.594^*^		0.512^*^
		7		0.489^*^		0.486^*^
		8		0.459^*^		
	Left	4		0.601^*^		0.674^*^
		9		0.422^*^		0.650^*^
BA9	Left	20				0.498^*^
		21		0.419^*^		
BA46	Right	1		0.476^*^		0.436^*^
BA8	Med.	25			0.464^*^	0.654^*^
	Left	26				0.617^*^
BA6	Med.	30		0.484^*^		
	Left	27		0.489^*^		0.528^*^
		29				0.650^*^
BA3	Med.	36	0.424^*^		0.442^*^	
BA7	Med.	40		0.410^*^		
	Left	44		0.406^*^		0.414^*^

* *Correlations of r < −0.4 were all significant at p < 0.01*.

### Correlations in the speed increase task

**Figure 5:**
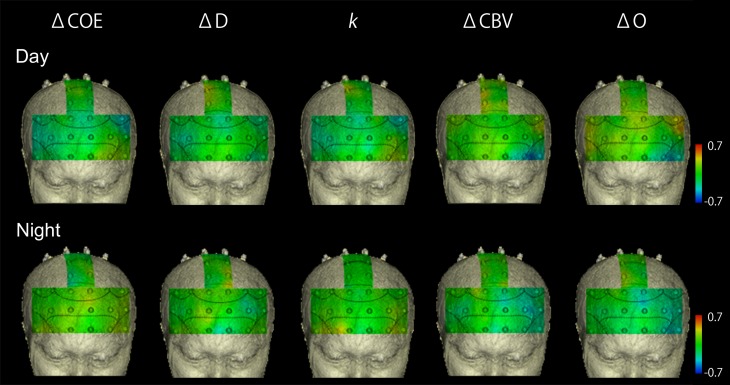
**Correlation maps showing correlations between vehicle acceleration and results from each of the hemodynamic indices in the speed increase task**. There were no striking correlations.

Figure 5 shows mapping images of the correlations between average vehicle acceleration and each of the hemodynamic indices in the speed increase task. Table 4 shows the correlations of 0.4 or more in this task.

**Table 4:** **Correlations in the speed increase task between vehicle acceleration and each of the brain activation indices (*p* < 0.01)**.

		**Ch**.	**ΔCOE**		**ΔCBV**		**Δ*O***	
			**Day**	**Night**	**Day**	**Night**	**Day**	**Night**
BA9	Left	16				−0.445^*^		
		20						−0.443^*^
		22	−0.416^*^				0.454^*^	
BA46	Left	5			−0.544^*^		−0.465^*^	−0.412^*^
BA7	Left	44				0.436^*^		0.461^*^

* *Correlations of r < −0.4 were all significant at p < 0.01*.

This task did not divide into positive and negative correlations depending on the index used. There were no correlation coefficients greater than ±0.5 in the speed increase task, except for ΔCBV in BA46. The only reproducible correlations found in either the daytime or nighttime tasks were in the left BA46. In the left BA46 (Ch. 5), Δ*O* demonstrated a negative correlation with vehicle acceleration in both the daytime and nighttime tasks (daytime: *r* = −0.465, *p* < 0.001; nighttime: *r* = −0.412, *p* < 0.001). The more rapid the acceleration, the more Δ*O* decreased in the left BA46.

### Interregional correlations with the supplementary eye field

**Figure 6:**
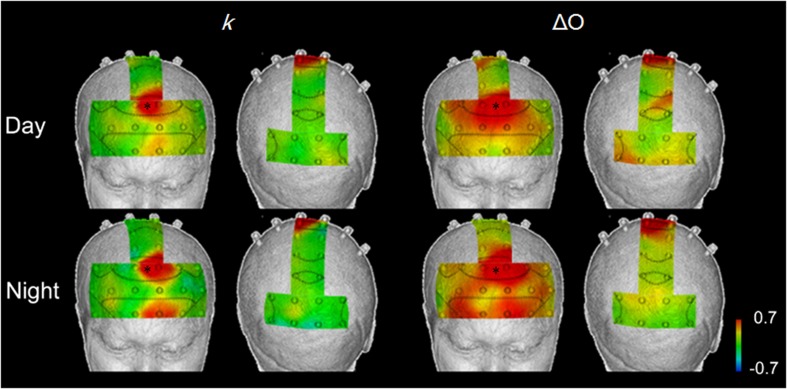
Correlation maps showing interregional correlations between responses in the supplementary eye field (^*^), where there were high correlations with vehicle acceleration during in both daytime and nighttime trials, and the responses in other areas, in the speed reduction task, using *k* and Δ*O*. *k* showed localized areas of correlation, while Δ*O* showed broad correlations in the prefrontal cortex.

Figure 6 shows mapping images of the correlations between responses in the supplementary eye field (medial BA8, where there was a high correlation with vehicle acceleration in both the daytime and nighttime tasks) and responses in the other areas, during the speed reduction task. Table 5 shows the number of channels with correlations of 0.4 or more with the medial BA8 (*r* > 0.4), by index.

**Table 5:** **Number of channels showing positive correlations with the medial BA8 (*r* > 0.4)**.

	***k***		**Δ*D***		**ΔCOE**		**ΔCBV**		**Δ*O***	
	**Day**	**Night**	**Day**	**Night**	**Day**	**Night**	**Day**	**Night**	**Day**	**Night**
BA10	2	3			3	6	2	5	5	6
BA9		1	5	1	6	6	9	6	8	6
BA46			1		1	1	1	3		2
BA8	1	1	1	1	2	1	2		2	2
BA6	1	1	4	1	2	1	6	3	3	4
BA4									1	
BA3					1				1	
BA7							2	1	2	
BA40								1	1	
Total	4	6	11	3	15	15	22	19	23	20

Interregional correlations with *k* were identified in 4–6 channels (daytime and nighttime tasks). For the other Hb indices, the number of correlations with the medial BA8 ranged from 11 to 23 channels. Particularly for Δ*O* and ΔCBV, localization was poor.

For *k* in the daytime speed reduction task, positive correlations with the medial BA8 were identified in the left BA8 (Ch. 26; *r* = 0.415), the left BA6 (Ch. 29; *r* = 0.657), the medial BA10 (Ch. 3; *r* = 0.415), and the left BA10 (Ch. 4; *r* = 0.446) (*p* < 0.01). These correlations were higher at night (0.477 < *r* < 0.741). Correlations also occurred in the area surrounding the left BA10 (Ch. 4; *r* = 0.630) and in BA9 (Ch. 20; *r* = 0.533) (*p* < 0.01).

## Discussion

### Relationship between changing vehicle speeds and brain activation

Correlations between the hemodynamic indices and vehicle acceleration were found to be higher in the speed reduction task, and lower in the speed increase task. In the speed reduction task, there were negative correlations between vehicle acceleration and the indices ΔCOE and *k* (that is, ΔCOE and *k* showed greater increases during faster deceleration). Because ΔCOE and *k* are indicators of change in oxygen metabolism, this suggests that oxygen metabolism increased during rapid deceleration of the vehicle.

Areas that typically exhibited greater increases in oxygen metabolism during vehicle speed reduction were BA8 and its surrounding area (including BA9 and BA6), which are involved in eye movement (Fukushima et al., 2000, 2002; Pierrot-Deseilligny et al., 2004). The field of view is narrower when the vehicle is driven at a high speed, while it spreads gradually, approaching a steady state when the vehicle speed decreases. BA8 controls side-to-side eye movements, and vergence eye movements responsible for depth perception (Gamlin and Yoon, 2000). The element of vergence eye movements is particularly important in fast vehicle traveling, but a more balanced ratio between the two types of eye movements would be required in slow vehicle traveling. This suggests that a spread in the direction of eye movement control occurs along with the spread in the field of view that occurs during rapid deceleration, and the activation of BA8 possibly increases at that time. In contrast, a possible reason for the low correlation between vehicle acceleration (positive acceleration) and the activation in BA8 during the vehicle speed increase task is that the acceleration of the vehicle results in a narrowing of the field of view, and the direction of the control direction easily becomes fixed in one direction.

Changes in vision enter the driver's brain as movement of the optic flow. It can be hypothesized that the increase in oxygen metabolism in BA8 results from the optic flow that is derived from changes in vehicle speed. The relationship between BA8 and the optic flow may be a key point in future research, as one of the regions of interest in the neuroimaging research on the driver's brain.

During nighttime driving, there were more correlations in the area surrounding BA8 than during daytime driving, and there were also more correlations between vehicle deceleration and oxygen metabolism in BA10, which is involved in executive function. During rapid deceleration at night, enhanced attention, or increased awareness of one's visual field and adjustments to one's eye movements may be more necessary than in the daytime. BA10 is known to be activated more in dual tasks (Baddeley and Della Sala, 1996). Vehicle deceleration at night requires control of the vehicle speed on a dark road without artificial lighting, and in addition, the useful field of view at night is narrower than in the daytime. It is thus likely that increased involvement of BA10 in deceleration at night occurs because the driver's attention must be allocated to a greater extent to both visual and motor control. Additional differences between daytime and nighttime driving may also exist in sites that were not included in our measurements.

### Interregional connectivity and the different brain activation indices

Interregional correlations between brain activation in BA8 and BA10 in both the daytime and at night were shown by the angle *k*. Correlations with vehicle acceleration, however, were observed in BA8 only during the day and in both BA8 and 10 at night. This suggests the possibility of a functional connectivity between BA8 and 10 regardless of the presence or absence of a correlation with vehicle acceleration. Anatomically, in addition to BA8, the frontal eye field also includes BA6 and BA9 (Goldberg et al., 1991). This could explain why correlations were higher with the areas surrounding BA8: BA6 (Ch. 29) and BA9 (Ch. 20). BA10 has been considered to be a separate area from BA8, and the detection of functional connectivity between these areas is thus a new finding. Further investigation of this point will be necessary.

The present study demonstrated that the identification of relationships between local brain activation differs according to the hemodynamic index used. Localized interregional relationships were shown by the angle *k* (degrees), which reflects variation in oxygen metabolism. The relationships shown by ΔCBV and Δ*O*, however, covered most of the prefrontal cortex. Hemodynamic responses in the blood vessels contain an oxygen metabolic component and a blood flow component. Differences in ΔCBV reflect the blood flow factor, and differences in ΔCOE reflects the oxygen metabolism factor. In other words, a hemodynamic index must include both indicators that reflect oxygen metabolic relationships and indicators that reflect vascular relationships. These factors are determined by the ratio of the variations in deoxyHb and oxyHb, and this is the ratio *k* on which the concept of phase is based. In the results of this study, the use of ΔCBV and Δ*O* reflected vascular relationships, and ΔCOE and *k* were likely reflected oxygen metabolic relationships.

To date, fNIRS studies have frequently been conducted using time series correlations of oxyHb to detect functional connectivity (Homae et al., 2010). Increases in oxyHb, however, are related to the inflow of arterial blood, and thus do not necessarily reflect increases in neural activity (Kato, 2004). Consequently, because these correlations based on oxyHb cannot distinguish actual activation from vascular networks in the scalp or the brain surface, the possibility that vascular relationships have been overestimated as functional connectivity cannot be denied. Skin blood flow are likely to be incorporated into measurements of oxyHb (Takahashi et al., 2011; Kirilina et al., 2012), and thus the use of phase, based on the angle *k*, has been proposed as a solution (Sano et al., 2013). In future research, the presence of differences in functional brain imaging based on these indices will need to be addressed as a technical and physiological issue.

Furthermore, in the present study we examined correlations using variation per second within the tasks, and this is another issue that it will be necessary to reconsider, with a view toward time series correlations in the future.

### The significance of neuroscience findings in ITS

Since research on the actual expressway driving is still in its infancy, the brain scientific findings are obviously still insufficient in the field of ITS as it relates to expressway construction and management. In particular, there is little basic knowledge about influences on the brain from the behavior of the vehicle in actual highway driving. There has been a simulation experiment on expressway driving using fMRI (Graydon et al., 2004), but it does not include the actual physical driving operations, and the field of view is different from that in actual road conditions. From the viewpoint of actual safety measures and expressway construction and management, the authors believe that the observation of brain activity in actual road experiments is essential.

As indicated in the introduction, it is a well-known fact that more accidents occur during deceleration than during acceleration, but the physiological reasons for this are not known. Our findings show that brain activation involved in voluntary eye movement control and in executive function is likely to increase while the driver is decelerating rapidly, especially at night. As these kinds of findings accumulate, it is possible that traffic safety measures can be developed that would help to increase or decrease brain activation during deceleration, by such means as redesign of deceleration lanes, or warning systems in places where sudden deceleration is likely to occur.

The correlations we found between prefrontal cortex activity and vehicle acceleration, do not necessarily imply a causal relationship. Time-course analysis will be required to clarify many issues in the relationship between the human brain and vehicle operation, including causality.

## Conclusion

This study demonstrated that prefrontal cortical activation increased with faster deceleration during actual road driving. This means that strong brain activation is required in situations when a driver has to brake rapidly. If the driver's prefrontal cortex does not work well during vehicle deceleration, the risk of accident may be increased. We also found that localized prefrontal cortical activation can be detected with good reproducibility by the simultaneous analysis of multiple hemodynamic indices with vector-based fNIRS, which makes it possible to detect both oxygen metabolic relationships and vascular relationships in the evaluation of brain activation.

### Conflict of interest statement

The authors declare that the research was conducted in the absence of any commercial or financial relationships that could be construed as a potential conflict of interest.
